# Supplementary material for: A novel RLIM/RNF12 variant disrupts protein stability and function to cause severe Tonne–Kalscheuer syndrome
Source: Sci Rep. 2021 May 5;11:9560. doi: 10.1038/s41598-021-88911-3 (PMC8100121; doi:10.1038/s41598-021-88911-3)

Supplementary Figure 1

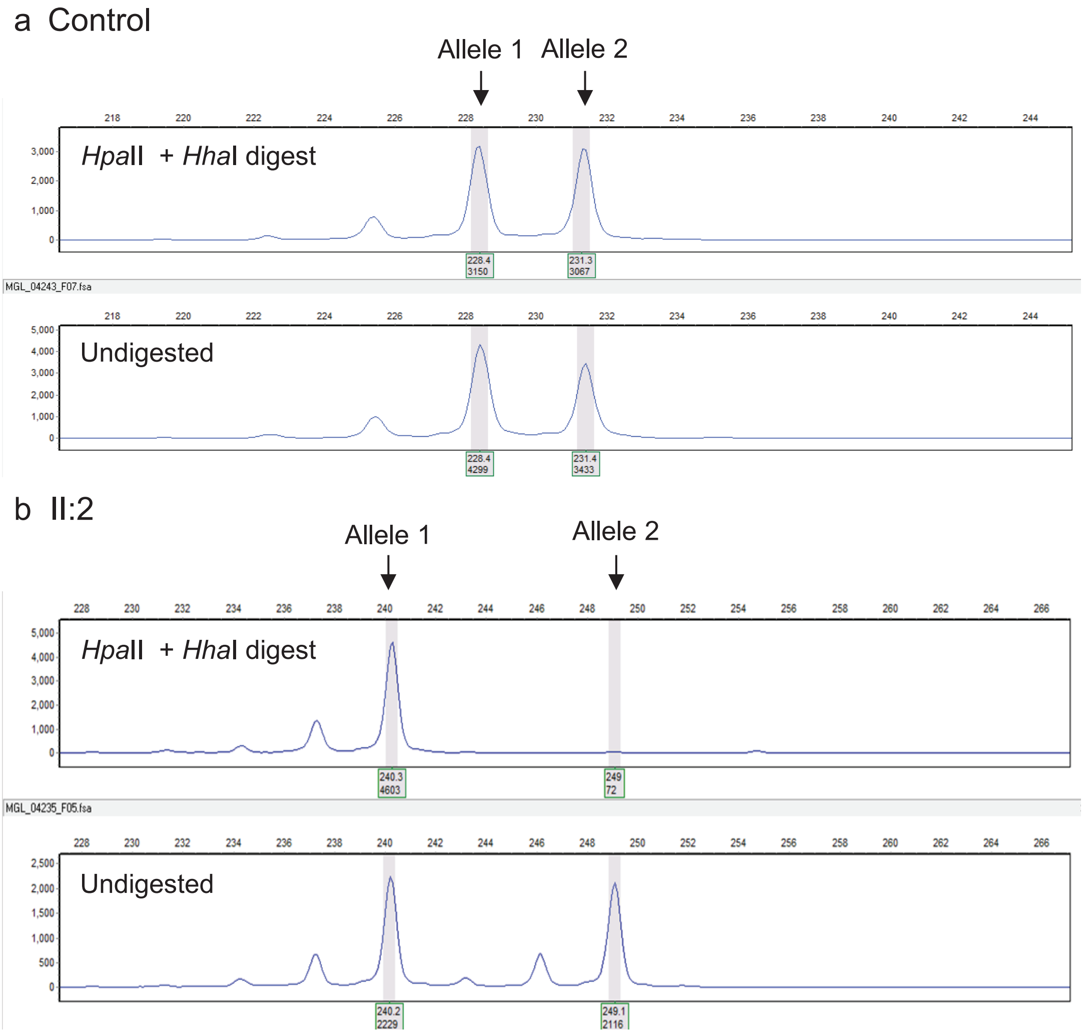

Subject	Fragment length (bp) allele 1	Fragment length (bp) allele 2	Cells with allele 1 as active X-chromosome (%)	Cells with allele 2 as active X-chromosome (%)
Control	228	231	50	50
II:2	240	249	5	95

Supplementary Figure 1: Skewed X-chromosome inactivation in the proband’s mother (II:2). DNA was subjected to methylation sensitive restriction enzyme digestion followed by PCR and fragment analysis. The inactive X-chromosome is not digested. Figure was generated using SoftGenetics GeneMarker v2.7 (https://softgenetics.com/GeneMarker.php).

Supplementary Figure 2

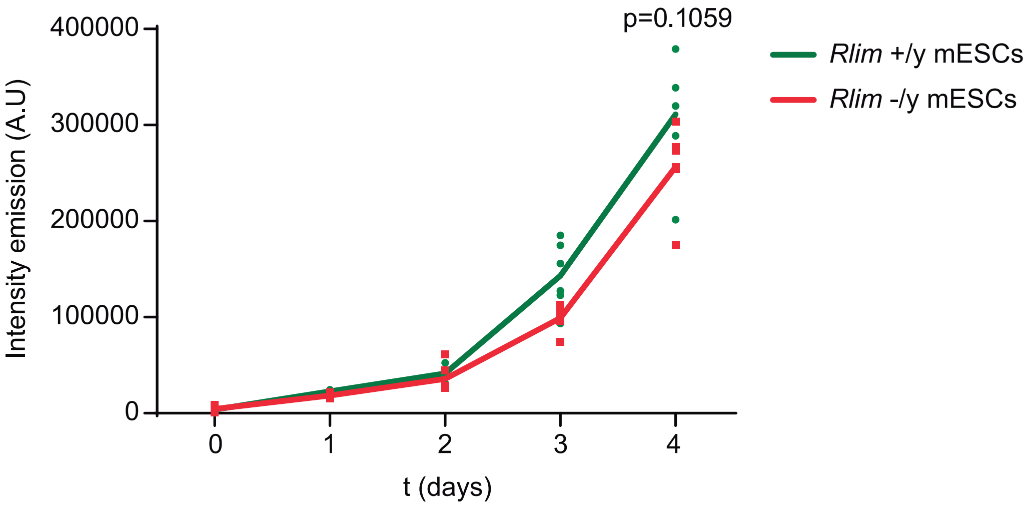

Supplementary Figure 2: Proliferation curves for indicated mESC lines cultured in LIF/FCS as determined by MTS assay. Data are represented as mean ± standard error of the mean (n=3). Statistical significance was determined by student's t-test. Figure was generated using Graphpad Prism v8.0 (https://www.graphpad.com/scientific-software/prism/).

Supplementary Figure 3: Unprocessed gels and blots

Figure 2a

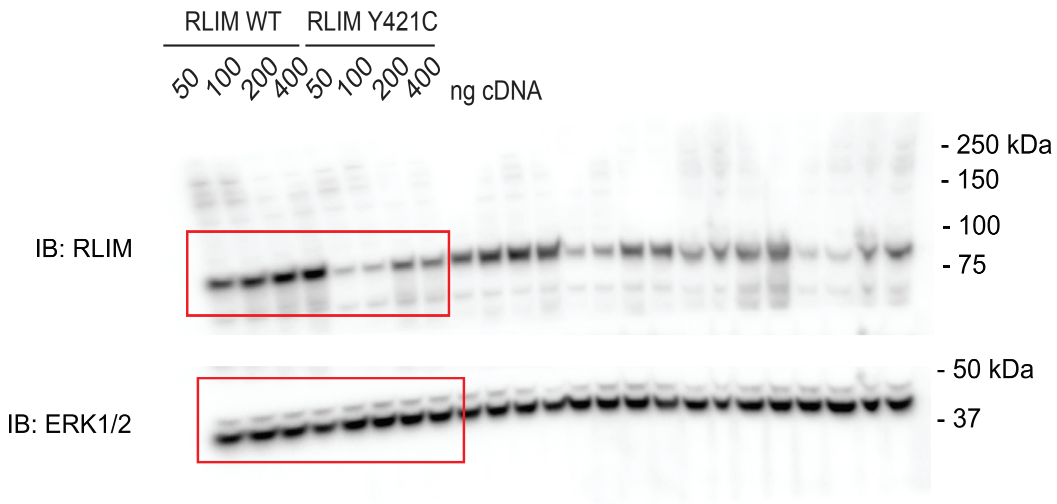

Figure 2c

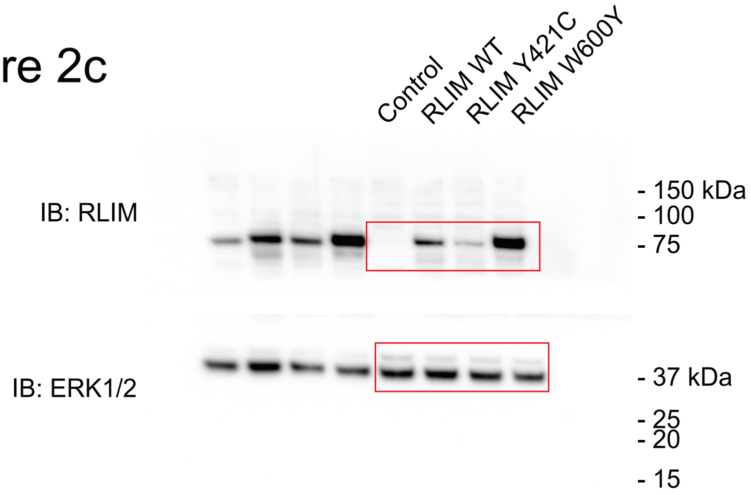

Figure 2c

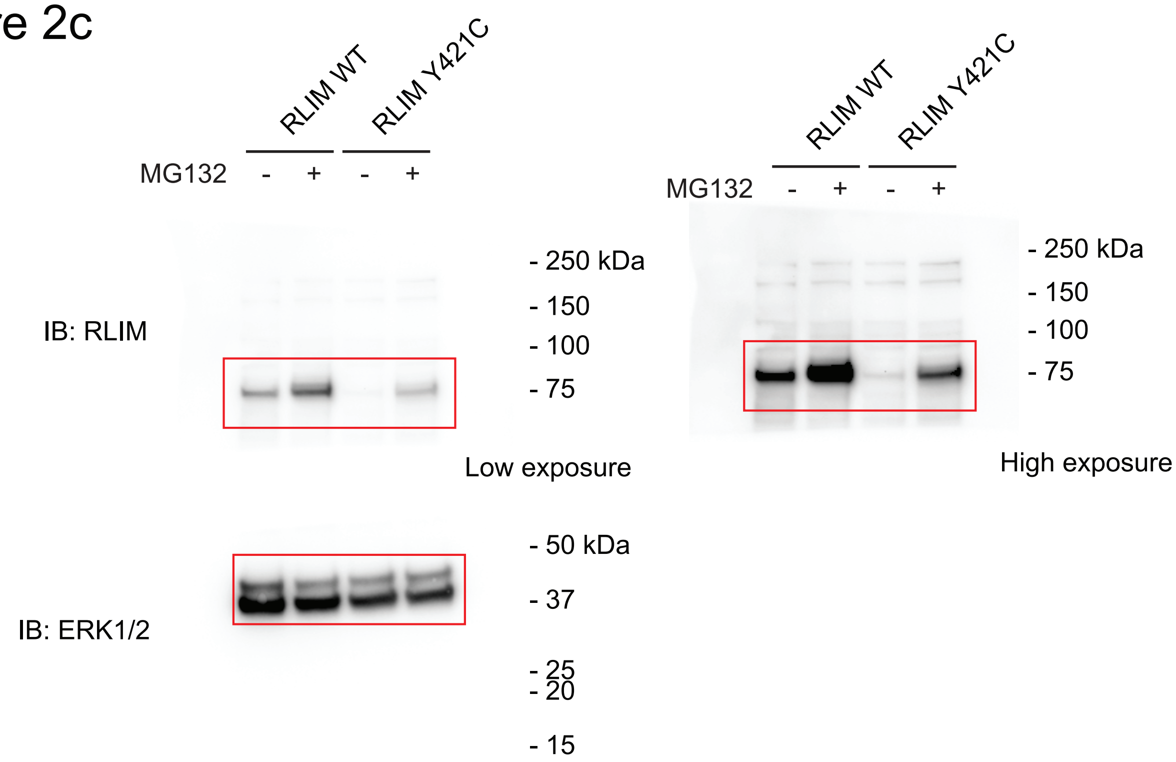

Figure 3a

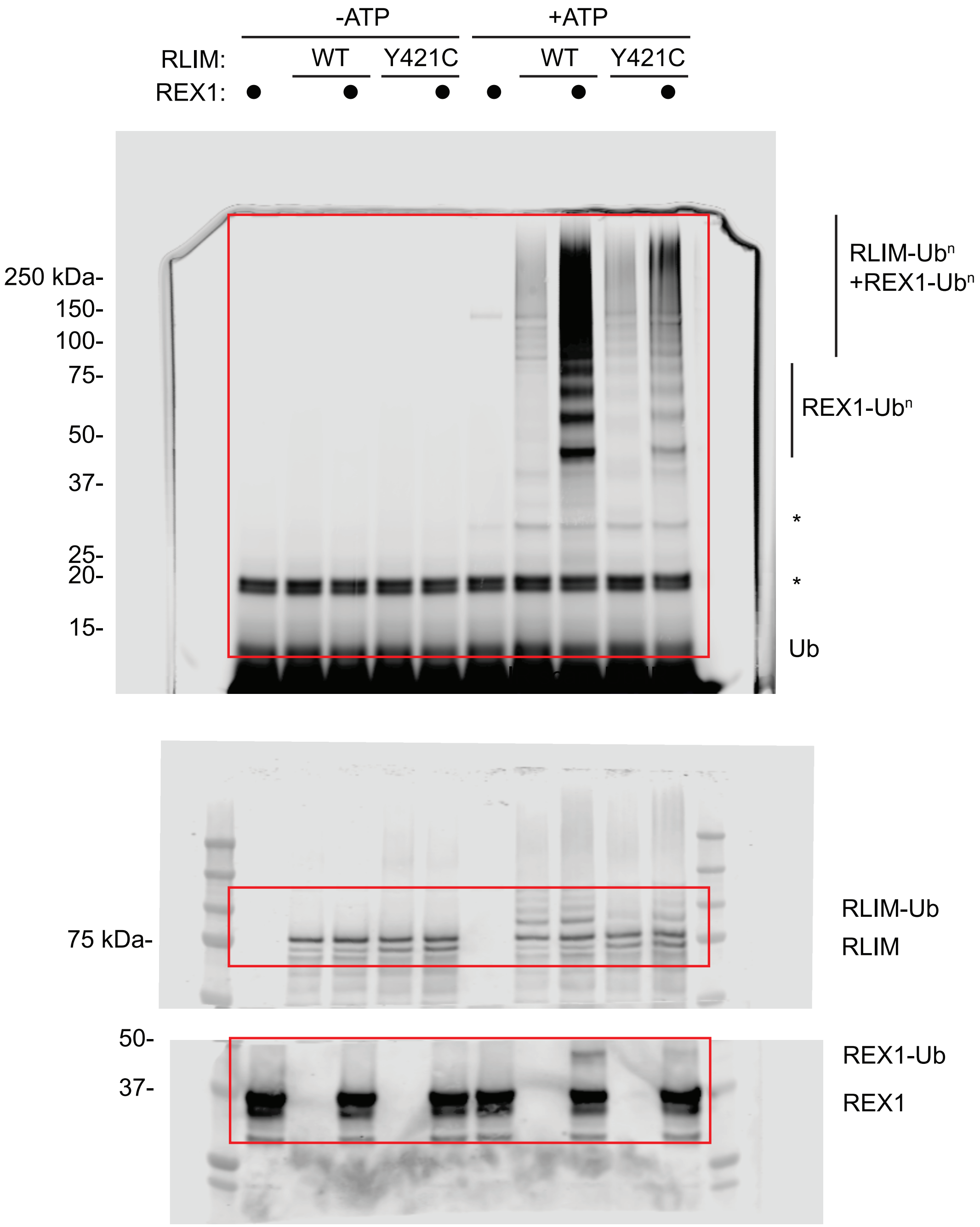

Figure 4b

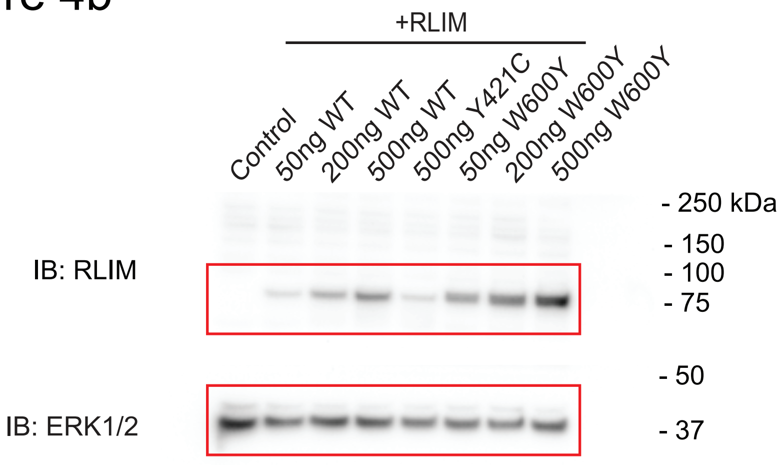

Supplement: Supplementary file 1 — Supplementary Information [file 41598_2021_88911_MOESM1_ESM.pdf]
